# Publisher Correction: SARS-CoV-2 Omicron is an immune escape variant with an altered cell entry pathway

**DOI:** 10.1038/s41564-022-01241-6

**Published:** 2022-09-16

**Authors:** Brian J. Willett, Joe Grove, Oscar A. MacLean, Craig Wilkie, Giuditta De Lorenzo, Wilhelm Furnon, Diego Cantoni, Sam Scott, Nicola Logan, Shirin Ashraf, Maria Manali, Agnieszka Szemiel, Vanessa Cowton, Elen Vink, William T. Harvey, Chris Davis, Patawee Asamaphan, Katherine Smollett, Lily Tong, Richard Orton, Joseph Hughes, Poppy Holland, Vanessa Silva, David J. Pascall, Kathryn Puxty, Ana da Silva Filipe, Gonzalo Yebra, Sharif Shaaban, Matthew T. G. Holden, Rute Maria Pinto, Rory Gunson, Kate Templeton, Pablo R. Murcia, Arvind H. Patel, Paul Klenerman, Susanna Dunachie, PITCH Consortium, Susanna Dunachie, Paul Klenerman, Eleanor Barnes, Anthony Brown, Sandra Adele, Barbara Kronsteiner, Sam M. Murray, Priyanka Abraham, Alexandra Deeks, M. Azim Ansari, Thushan de Silva, Lance Turtle, Shona Moore, James Austin, Alex Richter, Christopher Duncan, Rebecca Payne, The COVID-19 Genomics UK (COG-UK) Consortium, Amy Ash, Cherian Koshy, Beatrix Kele, Teresa Cutino-Moguel, Derek J. Fairley, James P. McKenna, Tanya Curran, Helen Adams, Christophe Fraser, David Bonsall, Helen Fryer, Katrina Lythgoe, Laura Thomson, Tanya Golubchik, Abigail Murray, Dawn Singleton, Shaun M. Beckwith, Anna Mantzouratou, Magdalena Barrow, Sarah L. Buchan, Nicola Reynolds, Ben Warne, Joshua Maksimovic, Karla Spellman, Kathryn McCluggage, Michaela John, Robert Beer, Safiah Afifi, Sian Morgan, Andrew Mack, Angela Marchbank, Anna Price, Arthur Morriss, Catherine Bresner, Christine Kitchen, Ian Merrick, Joel Southgate, Martyn Guest, Owen Jones, Robert Munn, Thomas R. Connor, Thomas Whalley, Trudy Workman, William Fuller, Amita Patel, Bindi Patel, Gaia Nebbia, Jonathan Edgeworth, Luke B. Snell, Rahul Batra, Themoula Charalampous, Angela H. Beckett, Ekaterina Shelest, Samuel C. Robson, Anthony P. Underwood, Ben E. W. Taylor, Corin A. Yeats, David M. Aanensen, Khalil Abudahab, Mirko Menegazzo, Amelia Joseph, Gemma Clark, Hannah C. Howson-Wells, Louise Berry, Manjinder Khakh, Michelle M. Lister, Tim Boswell, Vicki M. Fleming, Christopher W. Holmes, Claire L. McMurray, Jessica Shaw, Julian W. Tang, Karlie Fallon, Mina Odedra, Nicholas J. Willford, Paul W. Bird, Thomas Helmer, Lesley-Anne Williams, Nicola Sheriff, Sharon Campbell, Veena Raviprakash, Victoria Blakey, Christopher Moore, Fei Sang, Johnny Debebe, Matthew Carlile, Matthew W. Loose, Nadine Holmes, Victoria Wright, M. Estee Torok, William L. Hamilton, Alessandro M. Carabelli, Andrew Jermy, Beth Blane, Carol M. Churcher, Catherine Ludden, Dinesh Aggarwal, Elaine Westwick, Ellena Brooks, Georgina M. McManus, Katerina Galai, Ken Smith, Kim S. Smith, MacGregor Cox, Mireille Fragakis, Patrick Maxwell, Sarah Judges, Sharon J. Peacock, Theresa Feltwell, Anita Kenyon, Sahar Eldirdiri, Thomas Davis, Joshua F. Taylor, Ngee Keong Tan, Alex E. Zarebski, Bernardo Gutierrez, Jayna Raghwani, Louis du Plessis, Moritz U. G. Kraemer, Oliver G. Pybus, Sarah Francois, Stephen W. Attwood, Tetyana I. Vasylyeva, Aminu S. Jahun, Ian G. Goodfellow, Iliana Georgana, Malte L. Pinckert, Myra Hosmillo, Rhys Izuagbe, Yasmin Chaudhry, Felicity Ryan, Hannah Lowe, Samuel Moses, Luke Bedford, James S. Cargill, Warwick Hughes, Jonathan Moore, Susanne Stonehouse, Divya Shah, Jack C. D. Lee, Julianne R. Brown, Kathryn A. Harris, Laura Atkinson, Nathaniel Storey, Moira J. Spyer, Flavia Flaviani, Adela Alcolea-Medina, Jasveen Sehmi, John Ramble, Natasha Ohemeng-Kumi, Perminder Smith, Beatrice Bertolusso, Claire Thomas, Gabrielle Vernet, Jessica Lynch, Nathan Moore, Nicholas Cortes, Rebecca Williams, Stephen P. Kidd, Lisa J. Levett, Monika Pusok, Paul R. Grant, Stuart Kirk, Wendy Chatterton, Li Xu-McCrae, Darren L. Smith, Gregory R. Young, Matthew Bashton, Katie Kitchman, Kavitha Gajee, Kirstine Eastick, Patrick J. Lillie, Phillipa J. Burns, William Everson, Alison Cox, Alison H. Holmes, Frances Bolt, James R. Price, Marcus Pond, Paul A. Randell, Pinglawathee Madona, Siddharth Mookerjee, Erik M. Volz, Lily Geidelberg, Manon Ragonnet-Cronin, Olivia Boyd, Rob Johnson, Cassie F. Pope, Adam A. Witney, Irene M. Monahan, Kenneth G. Laing, Katherine L. Smollett, Alan McNally, Claire McMurray, Joanne Stockton, Joshua Quick, Nicholas J. Loman, Radoslaw Poplawski, Sam Nicholls, Will Rowe, Anibolina Castigador, Emily Macnaughton, Kate El Bouzidi, Malur Sudhanva, Temi Lampejo, Rocio T. Martinez Nunez, Cassie Breen, Graciela Sluga, Karen T. Withell, Nicholas W. Machin, Ryan P. George, Shazaad S. Y. Ahmad, David T. Pritchard, Debbie Binns, Nick Wong, Victoria James, Cheryl Williams, Chris J. Illingworth, Chris Jackson, Daniela de Angelis, David Pascall, Afrida Mukaddas, Alice Broos, Ana da Silva Filipe, Daniel Mair, David L. Robertson, Derek W. Wright, Emma C. Thomson, Igor Starinskij, Ioulia Tsatsani, James G. Shepherd, Jenna Nichols, Joseph Hughes, Kyriaki Nomikou, Lily Tong, Richard J. Orton, Sreenu Vattipally, William T. Harvey, Roy Sanderson, Sarah O’Brien, Steven Rushton, Jon Perkins, Rachel Blacow, Rory N. Gunson, Abbie Gallagher, Elizabeth Wastnedge, Kate E. Templeton, Martin P. McHugh, Rebecca Dewar, Seb Cotton, Lindsay Coupland, Rachael Stanley, Samir Dervisevic, Lewis G. Spurgin, Louise Smith, Clive Graham, Debra Padgett, Edward Barton, Garren Scott, Aidan Cross, Mariyam Mirfenderesky, Emma Swindells, Jane Greenaway, Rebecca Denton-Smith, Robyn Turnbull, Giles Idle, Kevin Cole, Amy Hollis, Andrew Nelson, Clare M. McCann, John H. Henderson, Matthew R. Crown, Wen C. Yew, William Stanley, Nichola Duckworth, Phillip Clarke, Sarah Walsh, Tim J. Sloan, Kelly Bicknell, Robert Impey, Sarah Wyllie, Scott Elliott, Sharon Glaysher, Declan T. Bradley, Nicholas F. Killough, Tim Wyatt, Andrew Bosworth, Barry B. Vipond, Clare Pearson, Elias Allara, Esther Robinson, Hannah M. Pymont, Husam Osman, Peter Muir, Richard Hopes, Stephanie Hutchings, Martin D. Curran, Surendra Parmar, Alicia Thornton, Angie Lackenby, Chloe Bishop, David Bibby, David Lee, Eileen Gallagher, Gavin Dabrera, Ian Harrison, Jonathan Hubb, Katherine A. Twohig, Meera Chand, Nicholas Ellaby, Nikos Manesis, Richard Myers, Steven Platt, Tamyo Mbisa, Vicki Chalker, Gonzalo Yebra, Matthew T. G. Holden, Sharif Shaaban, Stefan Rooke, Alec Birchley, Alexander Adams, Alisha Davies, Amy Gaskin, Bree Gatica-Wilcox, Caoimhe McKerr, Catherine Moore, Catryn Williams, Chris Williams, David Heyburn, Elen De Lacy, Ember Hilvers, Fatima Downing, Georgia Pugh, Hannah Jones, Hibo Asad, Jason Coombes, Jessica Hey, Jessica Powell, Joanne Watkins, Johnathan M. Evans, Laia Fina, Laura Gifford, Lauren Gilbert, Lee Graham, Malorie Perry, Mari Morgan, Matthew Bull, Nicole Pacchiarini, Noel Craine, Sally Corden, Sara Kumziene-Summerhayes, Sara Rey, Sarah Taylor, Simon Cottrell, Sophie Jones, Sue Edwards, Tara Annett, Alexander J. Trotter, Alison E. Mather, Alp Aydin, Andrew J. Page, David J. Baker, Ebenezer Foster-Nyarko, Gemma L. Kay, Justin O’Grady, Leonardo de Oliveira Martins, Lizzie Meadows, Nabil-Fareed Alikhan, Sophie J. Prosolek, Steven Rudder, Thanh Le-Viet, Anna Casey, Liz Ratcliffe, Aditi Singh, Arun Mariappan, Chris Baxter, Clara Radulescu, David A. Simpson, Deborah Lavin, Fiona Rogan, Julia Miskelly, Marc Fuchs, Miao Tang, Sílvia F. Carvalho, Stephen Bridgett, Timofey Skvortsov, Zoltan Molnar, Newara A. Ramadan, Bridget A. Knight, Christopher R. Jones, Cressida Auckland, Helen Morcrette, Jennifer Poyner, Dianne Irish-Tavares, Eric Witele, Jennifer Hart, Tabitha W. Mahungu, Tanzina Haque, Yann Bourgeois, Christopher Fearn, Kate F. Cook, Katie F. Loveson, Salman Goudarzi, Cariad Evans, David G. Partridge, Kate Johnson, Mehmet Yavus, Mohammad Raza, Craig Mower, Paul Baker, Sarah Essex, Stephen Bonner, Leanne J. Murray, Louisa K. Watson, Steven Liggett, Andrew I. Lawton, Ronan A. Lyons, Brendan A. I. Payne, Gary Eltringham, Jennifer Collins, Sheila Waugh, Shirelle Burton-Fanning, Yusri Taha, Christopher Jeanes, Andrea N. Gomes, Darren R. Murray, Maimuna Kimuli, Donald Dobie, Paula Ashfield, Angus Best, Benita Percival, Emma Moles-Garcia, Fiona Ashford, Jeremy Mirza, Liam Crawford, Megan Mayhew, Nicola Cumley, Oliver Megram, Dan Frampton, Judith Heaney, Matthew Byott, Catherine Houlihan, Charlotte A. Williams, Eleni Nastouli, Helen L. Lowe, John A. Hartley, Judith Breuer, Laurentiu Maftei, Leah Ensell, Marius Cotic, Matteo Mondani, Megan Driscoll, Nadua Bayzid, Rachel J. Williams, Sunando Roy, Adhyana I. K. Mahanama, Buddhini Samaraweera, Eleri Wilson-Davies, Emanuela Pelosi, Helen Umpleby, Helen Wheeler, Jacqui A. Prieto, Kordo Saeed, Matthew Harvey, Sarah Jeremiah, Siona Silviera, Stephen Aplin, Thea Sass, Ben Macklin, Dorian Crudgington, Liz A. Sheridan, Benjamin J. Cogger, Cassandra S. Malone, Florence Munemo, Hannah Huckson, Jonathan Lewis, Lisa J. Easton, Manasa Mutingwende, Michelle J. Erkiert, Mohammed O. Hassan-Ibrahim, Nicola J. Chaloner, Olga Podplomyk, Paul Randell, Roberto Nicodemi, Sarah Lowdon, Thomas Somassa, Alex Richter, Andrew Beggs, Andrew R. Hesketh, Colin P. Smith, Giselda Bucca, Chris Ruis, Claire Cormie, Ellen E. Higginson, Jamie Young, Joana Dias, Leanne M. Kermack, Mailis Maes, Ravi K. Gupta, Sally Forrest, Sophia T. Girgis, Rose K. Davidson, Áine O’Toole, Andrew Rambaut, Ben Jackson, Carlos E. Balcazar, Daniel Maloney, Emily Scher, J. T. McCrone, Kathleen A. Williamson, Michael D. Gallagher, Nathan Medd, Rachel Colquhoun, Thomas D. Stanton, Thomas Williams, Verity Hill, Aaron R. Jeffries, Ben Temperton, Christine M. Sambles, David J. Studholme, Joanna Warwick-Dugdale, Leigh M. Jackson, Michelle L. Michelsen, Robin Manley, Stephen L. Michell, Alistair C. Darby, Anita O. Lucaci, Charlotte Nelson, Claudia Wierzbicki, Edith E. Vamos, Hermione J. Webster, Kathryn A. Jackson, Lucille Rainbow, Margaret Hughes, Mark Whitehead, Matthew Gemmell, Miren Iturriza-Gomara, Richard Eccles, Richard Gregory, Sam T. Haldenby, Steve Paterson, Adrienn Angyal, Alexander J. Keeley, Benjamin H. Foulkes, Benjamin B. Lindsey, Dennis Wang, Hailey R. Hornsby, Luke R. Green, Manoj Pohare, Marta Gallis, Matthew D. Parker, Max Whiteley, Nikki Smith, Paige Wolverson, Peijun Zhang, Samantha E. Hansford, Sharon N. Hsu, Stavroula F. Louka, Thushan I. de Silva, Timothy M. Freeman, Matilde Mori, Emily J. Park, Jack D. Hill, Jayasree Dey, Jonathan Ball, Joseph G. Chappell, Patrick C. McClure, Timothy Byaruhanga, Arezou Fanaie, Geraldine Yaze, Rachel A. Hilson, Amy Trebes, Angie Green, David Buck, George MacIntyre-Cockett, John A. Todd, Andrew R. Bassett, Andrew Whitwham, Cordelia F. Langford, Diana Rajan, Dominic Kwiatkowski, Ewan M. Harrison, Iraad F. Bronner, Jaime M. Tovar-Corona, Jennifier Liddle, Jillian Durham, Katherine L. Bellis, Kevin Lewis, Louise Aigrain, Nicholas M. Redshaw, Robert M. Davies, Robin J. Moll, Shane A. McCarthy, Stefanie V. Lensing, Steven Leonard, Ben W. Farr, Carol Scott, Charlotte Beaver, Cristina V. Ariani, Danni Weldon, David K. Jackson, Emma Betteridge, Gerry Tonkin-Hill, Ian Johnston, Inigo Martincorena, James Bonfield, Jeffrey C. Barrett, John Sillitoe, Jon-Paul Keatley, Karen Oliver, Keith James, Lesley Shirley, Liam Prestwood, Luke Foulser, Marina Gourtovaia, Matthew J. Dorman, Michael A. Quail, Michael H. Spencer Chapman, Naomi R. Park, Rich Livett, Roberto Amato, Sally Kay, Scott Goodwin, Scott A. J. Thurston, Shavanthi Rajatileka, Sónia Gonçalves, Stephanie Lo, Theo Sanderson, Alasdair Maclean, Emily J. Goldstein, Lynne Ferguson, Rachael Tomb, Jana Catalan, Neil Jones, John Haughney, David L. Robertson, Massimo Palmarini, Surajit Ray, Emma C. Thomson

**Affiliations:** 1 MRC-University of Glasgow Centre for Virus Research, University of Glasgow, Glasgow, UK; 2 School of Mathematics & Statistics, University of Glasgow, Glasgow, UK; 3 NHS Greater Glasgow & Clyde, Glasgow, UK; 4 MRC Biostatistics Unit, University of Cambridge, Cambridge, UK; 5 Public Health Scotland, Glasgow, UK; 6 School of Medicine, University of St Andrews, St Andrews, UK; 7 NHS Lothian, Edinburgh, UK; 8 University of Oxford, Oxford, UK; 9 London School of Hygiene and Tropical Medicine, London, UK; 10 Peter Medawar Building for Pathogen Research, Nuffield Department of Clinical Medicine, University of Oxford, Oxford, UK; 11 Oxford University Hospitals NHS Foundation Trust, John Radcliffe Hospital, Oxford, UK; 12 Oxford Centre For Global Health Research, Nuffield Department of Clinical Medicine, University of Oxford, Oxford, UK; 13 Mahidol-Oxford Tropical Medicine Research Unit, Bangkok, Thailand; 14 Translational Gastroenterology Unit, University of Oxford, Oxford, UK; 15 NIHR Oxford Biomedical Research Centre, University of Oxford, Oxford, UK; 16 Department of Infection, Immunity and Cardiovascular Disease, University of Sheffield, Sheffield, UK; 17 Sheffield Teaching Hospitals NHS Foundation Trust, Sheffield, UK; 18 NIHR Health Protection Research Unit in Emerging and Zoonotic Infections, Institute of Infection, Veterinary and Ecological Sciences, University of Liverpool, Liverpool, UK; 19 Tropical and Infectious Disease Unit, Liverpool University Hospitals NHS Foundation Trust, Liverpool Health Partners, Liverpool, UK; 20 Institute of Cancer and Genomic Science, College of Medical and Dental Science, University of Birmingham, Birmingham, UK; 21 University Hospitals Birmingham NHS Foundation Trust, Birmingham, UK; 22 Translational and Clinical Research Institute Immunity and Inflammation Theme, Newcastle University, Newcastle, UK; 23 Department of Infection and Tropical Medicine, Newcastle upon Tyne Hospitals NHS Foundation Trust, Newcastle, UK; 24 Barking, Havering and Redbridge University Hospitals NHS Trust, Romford, UK; 25 Barts Health NHS Trust, London, UK; 26 Belfast Health and Social Care Trust, Belfast, UK; 27 Betsi Cadwaladr University Health Board, Bangor, UK; 28 Big Data Institute, Nuffield Department of Medicine, University of Oxford, Oxford, UK; 29 Blackpool Teaching Hospitals NHS Foundation Trust, Blackpool, UK; 30 Bournemouth University, Bournemouth, UK; 31 Cambridge Stem Cell Institute, University of Cambridge, Cambridge, UK; 32 Cambridge University Hospitals NHS Foundation Trust, Cambridge, UK; 33 Cardiff and Vale University Health Board, Cardiff, UK; 34 Cardiff University, Cardiff, UK; 35 Centre for Clinical Infection and Diagnostics Research, Department of Infectious Diseases, Guy’s and St Thomas’ NHS Foundation Trust, London, UK; 36 Centre for Enzyme Innovation, University of Portsmouth, Portsmouth, UK; 37 Centre for Genomic Pathogen Surveillance, University of Oxford, Oxford, UK; 38 Clinical Microbiology Department, Queens Medical Centre, Nottingham University Hospitals NHS Trust, Nottingham, UK; 39 Clinical Microbiology, University Hospitals of Leicester NHS Trust, Leicester, UK; 40 County Durham and Darlington NHS Foundation Trust, Darlington, UK; 41 Deep Seq, School of Life Sciences, Queens Medical Centre, University of Nottingham, Nottingham, UK; 42 Department of Infectious Diseases and Microbiology, Cambridge University Hospitals NHS Foundation Trust, Cambridge, UK; 43 Department of Medicine, University of Cambridge, Cambridge, UK; 44 Department of Microbiology, Kettering General Hospital, Kettering, UK; 45 Department of Microbiology, South West London Pathology, London, UK; 46 Department of Zoology, University of Oxford, Oxford, UK; 47 Division of Virology, Department of Pathology, University of Cambridge, Cambridge, UK; 48 East Kent Hospitals University NHS Foundation Trust, Canterbury, UK; 49 East Suffolk and North Essex NHS Foundation Trust, Colchester, UK; 50 East Sussex Healthcare NHS Trust, St. Leonards-on-Sea, UK; 51 Gateshead Health NHS Foundation Trust, Gateshead, UK; 52 Great Ormond Street Hospital for Children NHS Foundation Trust, London, UK; 53 Great Ormond Street Institute of Child Health (GOS ICH), University College London (UCL), London, UK; 54 Guy’s and St. Thomas’ Biomedical Research Centre, London, UK; 55 Guy’s and St. Thomas’ NHS Foundation Trust, London, UK; 56 Hampshire Hospitals NHS Foundation Trust, Basingstoke, UK; 57 Health Services Laboratories, London, UK; 58 Heartlands Hospital, Birmingham, Birmingham, UK; 59 Hub for Biotechnology in the Built Environment, Northumbria University, Newcastle upon Tyne, UK; 60 Hull University Teaching Hospitals NHS Trust, Hull, UK; 61 Imperial College Healthcare NHS Trust, London, UK; 62 Imperial College London, London, UK; 63 Infection Care Group, St George’s University Hospitals NHS Foundation Trust, London, UK; 64 Institute for Infection and Immunity, St George’s University of London, London, UK; 65 Institute of Biodiversity, Animal Health & Comparative Medicine, University of Glasgow, Glasgow, UK; 66 Institute of Microbiology and Infection, University of Birmingham, Birmingham, UK; 67 Isle of Wight NHS Trust, Newport, UK; 68 King’s College Hospital NHS Foundation Trust, London, UK; 69 King’s College London, London, UK; 70 Liverpool Clinical Laboratories, Liverpool, UK; 71 Maidstone and Tunbridge Wells NHS Trust, Maidstone, UK; 72 Manchester University NHS Foundation Trust, Manchester, UK; 73 Microbiology Department, Buckinghamshire Healthcare NHS Trust, Aylesbury, UK; 74 Microbiology Department, Buckinghamshire Healthcare NHS Trust, Aylesbury, UK; 75 Microbiology, Royal Oldham Hospital, Oldham, UK; 76 MRC Biostatistics Unit, University of Cambridge, Cambridge, UK; 77 MRC-University of Glasgow Centre for Virus Research, Glasgow, UK; 78 Newcastle University, Newcastle upon Tyne, UK; 79 NHS Greater Glasgow and Clyde, Glasgow, UK; 80 NHS Lothian, Edinburgh, UK; 81 Norfolk and Norwich University Hospitals NHS Foundation Trust, Norwich, UK; 82 Norfolk County Council, Norwich, UK; 83 North Cumbria Integrated Care NHS Foundation Trust, Carlisle, UK; 84 North Middlesex University Hospital NHS Trust, London, UK; 85 North Tees and Hartlepool NHS Foundation Trust, Stockton on Tees, UK; 86 Northumbria Healthcare NHS Foundation Trust, Newcastle upon Tyne, UK; 87 Northumbria University, Newcastle upon Tyne, UK; 88 Path Links, Northern Lincolnshire and Goole NHS Foundation Trust, Grimsby, UK; 89 Portsmouth Hospitals University NHS Trust, Portsmouth, UK; 90 Public Health Agency, Northern Ireland, Belfast, UK; 91 Public Health England, London, UK; 92 Public Health England, Cambridge, Cambridge, UK; 93 Public Health England, Colindale, London, UK; 94 Public Health Scotland, Edinburgh, UK; 95 Public Health Wales, Cardiff, UK; 96 Quadram Institute Bioscience, Norwich, UK; 97 Queen Elizabeth Hospital, Birmingham, Birmingham, UK; 98 Queen’s University Belfast, Belfast, UK; 99 Royal Brompton and Harefield Hospitals, London, UK; 100 Royal Devon and Exeter NHS Foundation Trust, Exeter, UK; 101 Royal Free London NHS Foundation Trust, London, UK; 102 School of Biological Sciences, University of Portsmouth, Portsmouth, UK; 103 School of Pharmacy & Biomedical Sciences, University of Portsmouth, Portsmouth, UK; 104 Sheffield Teaching Hospitals NHS Foundation Trust, Sheffield, UK; 105 South Tees Hospitals NHS Foundation Trust, Middlesbrough, UK; 106 Southwest Pathology Services, Taunton, UK; 107 Swansea University, Swansea, UK; 108 The Newcastle upon Tyne Hospitals NHS Foundation Trust, Newcastle upon Tyne, UK; 109 The Queen Elizabeth Hospital King’s Lynn NHS Foundation Trust, King’s Lynn, UK; 110 The Royal Marsden NHS Foundation Trust, London, UK; 111 The Royal Wolverhampton NHS Trust, Wolverhampton, UK; 112 Turnkey Laboratory, University of Birmingham, Birmingham, UK; 113 University College London Division of Infection and Immunity, London, UK; 114 University College London Hospital Advanced Pathogen Diagnostics Unit, London, UK; 115 University College London Hospitals NHS Foundation Trust, London, UK; 116 University Hospital Southampton NHS Foundation Trust, Southampton, UK; 117 University Hospitals Dorset NHS Foundation Trust, Poole, UK; 118 University Hospitals Sussex NHS Foundation Trust, Worthing, UK; 119 University of Birmingham, Birmingham, UK; 120 University of Brighton, Brighton, UK; 121 University of Cambridge, Cambridge, UK; 122 University of East Anglia, Norwich, UK; 123 University of Edinburgh, Edinburgh, UK; 124 University of Exeter, Exeter, UK; 125 University of Liverpool, Liverpool, UK; 126 University of Sheffield, Sheffield, UK; 127 University of Southampton, Nottingham, UK; 128 Virology, School of Life Sciences, Queens Medical Centre, University of Nottingham, Nottingham, UK; 129 Watford General Hospital, Watford, UK; 130 Wellcome Centre for Human Genetics, Nuffield Department of Medicine, University of Oxford, Oxford, UK; 131 Wellcome Sanger Institute, Hinxton, UK; 132 West of Scotland Specialist Virology Centre, NHS Greater Glasgow and Clyde, Glasgow, UK; 133 Whittington Health NHS Trust, London, UK

**Keywords:** Molecular biology, Immunology, Viral infection, Vaccines

Correction to: *Nature Microbiology* 10.1038/s41564-022-01143-7, published online 7 July 2022.

In the version of this article initially published, the author affiliation information was incomplete, neglecting to note that Brian J. Willett, Joe Grove, Oscar A. MacLean, Craig Wilkie, Giuditta De Lorenzo, Wilhelm Furnon, Diego Cantoni, Sam Scott, Nicola Logan and Shirin Ashraf contributed equally and that John Haughney, David L. Robertson, Massimo Palmarini, Surajit Ray and Emma C. Thomson jointly supervised the work, as now indicated in the HTML and PDF versions of the article.

